# Increased detection of primary carnitine deficiency through second-tier newborn genetic screening

**DOI:** 10.1186/s13023-021-01785-6

**Published:** 2021-03-23

**Authors:** Yiming Lin, Weifeng Zhang, Chenggang Huang, Chunmei Lin, Weihua Lin, Weilin Peng, Qingliu Fu, Dongmei Chen

**Affiliations:** 1 Neonatal Disease Screening Center, Quanzhou Maternity and Children’s Hospital, 700 Fengze Street, Quanzhou, 362000 Fujian Province China; 2 Department of Neonatal Intensive Care Unit, Quanzhou Maternity and Children’s Hospital, 700 Fengze Street, Quanzhou, 362000 Fujian Province China; 3 Zhejiang Biosan Biochemical Technologies Co., Ltd, Hangzhou, China

**Keywords:** Primary carnitine deficiency, Newborn screening, Free carnitine, Second-tier screening

## Abstract

**Background:**

Newborn screening for primary carnitine deficiency (NBS) is commonly implemented worldwide; however, it has poor sensitivity. This study aimed to evaluate the feasibility of improving screening by including a second-tier genetic assay.

**Results:**

An Agena iPLEX assay was developed to identify 17 common *SLC22A5* mutations in Chinese populations and was applied in NBS as a second-tier screening. From January 2017 to December 2018, 204,777 newborns were screened for PCD using tandem mass spectrometry. A total of 316 (0.15%) residual NBS-positive specimens with low free carnitine (C0) levels were subjected to this second-tier screening. The screening identified 20 screen-positive newborns who harboured biallelic mutations in the*SLC22A5* gene, 99 carriers with one mutation, and 197 screen-negative newborns with no mutations. Among the 99 carriers, four newborns were found to have a second disease-causing *SLC22A5*mutation by further genetic analysis. Among the 197 screen-negatives were four newborns with persistently low C0 levels, and further genetic analysis revealed that one newborn had two novel *SLC22A5* pathogenic variants. In total, 25 newborns were diagnosed with PCD, for a positive predictive value of 7.91% (25/316). Based on these data, we estimate the incidence of PCD in Quanzhou is estimated to be 1:8191.Thirteen distinct *SLC22A5* variants were identified, and the most common was c.760C > T, with an allelic frequency of 32% (16/50), followed by c.1400C > G (7/50, 14%), and c.51C > G (7/50, 14%).

**Conclusion:**

Data from this study revealed that 24% (6/25) of PCD cases would have been missed by conventional NBS. This high-throughput iPLEX assay is a powerful tool for PCD genotyping. The addition of this second-tier genetic screening to the current NBS program could identify missed PCD cases, thereby increasing PCD detection. However, further studies are needed to optimise the workflow of the new screening algorithm and to evaluate the cost-effectiveness of this screening approach.

**Supplementary Information:**

The online version contains supplementary material available at 10.1186/s13023-021-01785-6.

## Background

Primary carnitine deficiency (PCD, OMIM #212140) is an autosomal recessive disorder of fatty acid oxidation caused by biallelic mutations in the *SLC22A5* gene, which encodes organic cation transporter type 2 (OCTN2) [1–3]. This defect leads to urinary carnitine wasting, low serum carnitine levels, and decreased intracellular carnitine accumulation. The clinical presentation of PCD is highly variable, ranging from no clinical symptoms to hypoketotic hypoglycaemia and hepatic encephalopathy early in life, progressive hypertrophic cardiomyopathy later in life, or even sudden death from cardiac arrhythmia [4–6]. Although some patients appear to be asymptomatic, PCD is a potentially lethal disease, and recent studies have shown that untreated PCD is associated with sudden death [7, 8]. Fortunately, with timely treatment, the long-term prognosis is favourable, as most symptoms are reversible [9].

Given the potential severity of PCD and the availability of effective treatment, PCD is included in many newborn screening (NBS) programs worldwide [10–12]. Tandem mass spectrometry (MS/MS) is mainly used in NBS for PCD, which can be detected by measuring free carnitine (C0) levels in a dried blood spot (DBS). However, the current MS/MS-based PCD NBS has poor sensitivity, leading to some neonates passing the NBS, yet presenting with cardiac failure and dilated cardiomyopathy due to PCD [13–17]. Recently, the New Zealand NBS program discontinued PCD NBS because of its low sensitivity [14]. Therefore, additional or second-tier testing is required to improve the performance of PCD NBS.

The Agena iPLEX assay is an effective and reliable approach for mutation screening, and our previous work demonstrated the feasibility of incorporating genetic screening into the current NBS program [18, 19]. Here, an Agena iPLEX PCD assay was developed to identify 17 common *SLC22A5* mutations in the Chinese population, which was then applied as a second-tier test to improve the performance of PCD NBS. This study presents the results of the implementation of this new screening algorithm.

## Results

### Assay validation

A double-blind analysis of 20PCD-positive DBS samples was conducted to validate the robustness of the iPLEX genotyping assay. The iPLEX assay correctly detected all *SLC22A5* mutations in the 20 samples, indicating its high sensitivity and specificity (Table 1).

**Table 1:** Genotypes of 20 patients with PCD detected by the iPLEX assay

No	Genotype	iPLEX NICCD assay	Targeted NGS
1	c.760C > T/c.1400C > G	6	6
2	c.760C > T/c.760C > T	3	3
3	c.51C > G/c.760C > T	2	2
4	c.51C > G/c.51C > G	2	2
5	c.760C > T/c.797C > T	2	2
6	c.695C > T/c.1139C > T	2	2
7	c.51C > G/c.338G > A	1	1
8	c.51C > G/c.1195C > T	1	1
9	c.517delC/c.797C > T	1	1
Total		20	20

### Newborn genetic screening and diagnosis

Overall, 316 (0.15%) of the 204,777 newborns screened via MS/MS at Quanzhou Maternity and Children’s Hospital had C0 low levels on the first screen and underwent second-tier genetic screening. The results of the second-tier testing identified 20 screen-positive newborns who harboured biallelic mutations in the*SLC22A5* gene, 99 carriers with one mutation, and 197 screen-negative newborns with no mutations. Further genetic testing showed that 4 of the 99 carriers had a second *SLC22A5* mutation. All 197 screen-negative newborns were retested using MS/MS, and four of them with persistently low C0 levels in this second screen underwent further genetic testing. This testing revealed one newborn with two novel *SLC22A5* pathogenic variants. In total, 25 newborns were diagnosed with PCD by genetic analyses, for a positive predictive value of 7.91% (25/316) (Table 2). Therefore, during the study period, the incidence of PCD in the selected population was estimated to be 1:8191.

**Table 2:** Biochemical and genetic characteristics of patients with PCD

No	Gender	NC0 levels (μmol/L)	RC0 levels (μmol/L)	Screened by MassArray assay		Confirmed by NGS	
				Allele 1	Allele 2	Allele 1	Allele 2
1	Male	1.96	1.73	c.760C > T	c.51C > G	c.760C > T	c.51C > G
2	Female	2.40	1.44	c.760C > T	c.760C > T	c.760C > T	c.760C > T
3	Male	5.78	10.67	c.760C > T	c.797C > T	c.760C > T	c.797C > T
4	Male	5.95	8.64	c.695C > T	NF	c.695C > T	**c.1160A > G**
5	Female	7.27	6.66	c.760C > T	c.797C > T	c.760C > T	c.797C > T
6	Female	5.58	5.59	c.760C > T	c.1400C > G	c.760C > T	c.1400C > G
7	Female	5.34	6.02	c.797C > T	NF	c.797C > T	**c.394-1G > A**
8	Female	1.78	1.90	c.695C > T	c.1139C > T	c.695C > T	c.1139C > T
9	Male	4.34	4.45	c.51C > G	c.51C > G	c.51C > G	c.51C > G
10	Female	4.75	4.16	c.760C > T	NF	c.760C > T	c.845G > A
11	Female	3.45	5.24	c.760C > T	c.1400C > G	c.760C > T	c.1400C > G
12	Female	6.82	5.02	c.760C > T	c.1400C > G	c.760C > T	c.1400C > G
13	Male	2.19	2.12	NF	NF	**c.822G > A**	**c.782_799del**
14	Male	2.73	9.84	c.51C > G	NF	c.51C > G	**c.1144_1162del**
15	Male	3.00	10.81	c.51C > G	c.1400C > G	c.51C > G	c.1400C > G
16	Male	6.46	5.10	c.695C > T	c.1400C > G	c.695C > T	c.1400C > G
17	Male	3.02	1.77	c.760C > T	c.760C > T	c.760C > T	c.760C > T
18	Female	6.77	10.05	c.1400C > G	c.1400C > G	c.1400C > G	c.1400C > G
19	Female	2.36	1.75	c.760C > T	c.760C > T	c.760C > T	c.760C > T
20	Female	3.12	2.88	c.760C > T	c.51C > G	c.760C > T	c.51C > G
21	Male	3.64	3.80	c.695C > T	c.1139C > T	c.695C > T	c.1139C > T
22	Female	3.56	4.31	c.760C > T	c.1139C > T	c.760C > T	c.1139C > T
23	Female	6.27	3.43	c.695C > T	c.1139C > T	c.695C > T	c.1139C > T
24	Female	2.70	3.46	c.760C > T	c.51C > G	c.760C > T	c.51C > G
25	Male	7.35	14.27	c.338G > A	c.338G > A	c.338G > A	c.338G > A

NC0: free carnitine detected at newborn screening, RC0: C0 retested at recall stage, cutoff value: 8–50 μmol/L

The novel *SLC22A5* variants identified by our team are in boldface type

*NF* not found

Thirteen distinct *SLC22A5* variants were identified, seven of which were included in the designed panel, one previously reported mutation, c.845G > A, was not included in the designed panel, and the remaining five were newly identified pathogenic variants that were recently reported by our team [20].The most common mutation was c.760C > T, with an allelic frequency of 32% (16/50), followed by c.1400C > G (7/50, 14%), and c.51C > G (7/50, 14%). Four other mutations, c.695C > T, c.1139C > T, c.338G > A, and c.797C > T were also relatively common mutations. These seven mutations accounted for 88% (44/50) of the detected mutant alleles (Table 3).

**Table 3:** Detected *SLC22A5* variants and their frequencies

No	Variants	Alleles	Frequencies (%)
1	760C > T	16	32
2	c.1400C > G	7	14
3	c.51C > G	7	14
4	c.695C > T	5	10
5	c.1139C > T	4	8
6	c.797C > T	3	6
7	c.338G > A	2	4
8	c.845G > A	1	2
9	**c.394-1G > A**	1	2
10	**c.782_799del**	1	2
11	**c.822G > A**	1	2
12	**c.1144_1162del**	1	2
13	**c.1160A > G**	1	2

The novel *SLC22A5* variants identified by our team are in boldface type

### Biochemical characteristics

The mean C0 concentration at NBS in this cohort was 4.34 ± 1.81 μmol/L. At recall, the mean C0 concentration was increased to 5.40 ± 2.94 μmol/Lin the second screening. Of note, six patients (no. 3, 4, 14, 15, 18, and 25) with low C0 levels (2.73–7.35 μmol/L) in the first screening had C0 levels (8.64–14.27 μmol/L) that were above the normal cut-off value in the second screening (Table 2).

## Discussion

The sensitivity of NBS for PCD is unsatisfactory because a small proportion of PCD patients are missed using the current MS/MS-based screening approach. Our findings revealed a proportion of cases (24%, 6/25) that were missed by conventional NBS. Therefore, physicians should note that a normal C0 level during recall does not rule out PCD. Incorporating this second-tier mutation test into the current NBS program would introduce an opportunity to identify missed PCD cases. The high-throughput iPLEX genotyping assay that was used is low-cost (~ $3.6/sample) and has a fast turn-around time of 7 h (from DNA preparation to data report), and it was proven to be robust and reliable in the validation. Six PCD patients who would have been missed were successfully identified when we applied the iPLEX assay as a second-tier screening method. Therefore, we clearly demonstrated that incorporating second-tier molecular genetic testing into the current NBS program could increase PCD detection.

The iPLEX assay has several advantages. First, as we demonstrated in our previous work, it is a powerful tool for population-based genetic screening [18, 19]. In this study, the iPLEX assay was applied in NBS for second-tier screening of suspected PCD, which should provide rapid diagnosis of most PCD patients and help shorten the time to diagnosis. The application of this second-tier testing led to early identification of six patients with PCD (no. 3, 4, 14, 15, 18, and 25), which enabled them to receive timely treatment and prevent the occurrence of adverse symptoms. These six patients would been excluded based on their normal results in the second test of a conventional NBS, which means that these patients would have escaped detection if the new screening algorithm was not implemented, indicating the importance of this second-tier genetic testing for discovering latent PCD patients in the NBS program. However, it is noteworthy that the improvement in sensitivity comes at the expense of increased carrier identification. A previous study in Taiwan used second-tier molecular tests to screen for c.760C > T in 206 newborns with low C0 levels and found 10 carriers, which were excluded due to their normal C0 levels in the second screen [21]. In contrast, our study found that 4% (4/99) of carriers had a second *SLC22A5*mutation/variant and were diagnosed with PCD (no. 4, 7, 10, and 14) rather than as carriers, and two of them had normal C0 levels in the second screening. The challenge is how best to use this assay to increase detection and minimise the detection of unaffected carriers that require further genetic analysis. Thus, large-scale studies are needed to optimise the workflow of the second-tier genetic screening and to evaluate the cost-effectiveness of this screening approach. Notably, although the panel with 17 hotspots was designed to detect the majority of *SLC22A5* mutations in China, one patient (no. 13) with extremely low C0 levels was missed by our second-tier genetic testing. All predefined panels are faced with this deficiency because the known mutations in a specific population have been less well studied. Due to the extremely low C0 levels of this study subject in the second screening, NGS was performed, and two novel *SLC22A5* pathogenic variants were identified. Therefore, further genetic analysis is required when newborns have persistently low C0 levels, but no mutation is found in the second-tier genetic screening.

Based on NBS including second-tier genetic screening, the incidence of PCD in the selected population was estimated at 1:8191. This incidence is higher than that reported in most other regions of China [9, 22, 23]. Although there may be regional differences, our findings suggest that the true incidence of PCD in China might be underestimated. Consistent with many previous studies [24, 25], c.760C > T (p.R254*) was the most frequently occurring mutation in this cohort. This mutation along with two others, c.1400C > G and c.51C > G, had a combined frequency of 60%, confirming that these are hotspot mutations among Chinese populations [24, 26].

This study had some limitations. First, the sole criterion for inclusion was an abnormal PCD NBS result. However, it is important to note that C0 levels in newborns can be affected by the mother because C0 is transported to the foetus via the placenta [6, 12, 27]. Using the current algorithm, a newborn with PCD with a false-negative result of normal C0 levels in the NBS would not be selected for second-tier *SLC22A5* mutation analysis. Therefore, it is possible that some PCD cases missed during NBS would not have come to our attention. Utilising a higher C0 cut-off value could reduce the number of missed cases during NBS; however, this may result in an unacceptable percentage of false positives. Balancing these two metrics will be challenging. Second, 193 of the 197 screen-negative newborns were excluded due to normal C0 levels in the second screening, and it is possible that one or more of these 193 newborns classified as screen-negative might have PCD, although the likelihood is very small. However, given that the cost and the fact that the second-tier genetic screening did not detect any *SLC22A5* mutations, no further genetic analysis was performed to verify the results. Third, the iPLEX assay can only identify known mutations in the designed assay panel. Our designed panel, with 17 hotspots, can only detect the majority of*SLC22A5* mutations in the Chinese population, and untargeted variants will be missed, as demonstrated by the finding of two novel *SLC22A5* variants and a previously reported mutation in this study.

## Conclusions

In summary, the incidence of PCD is relatively high in Quanzhou, China. Data from this study revealed that 24% (6/25) of PCD cases identified by our genetic screen would have been missed by conventional NBS. The high-throughput iPLEX assay is a powerful tool for PCD genotyping. The incorporation of second-tier genetic screening into the current PCD NBS program could introduce an opportunity to identify missed PCD cases, thereby increasing PCD detection. PCD NBS continues to be a challenge, as newborns with persistently low C0 levels would require combined genetic analyses, and further studies are needed to optimise the workflow of the new screening algorithm and evaluate the cost-effectiveness of this screening approach.

## Methods

### Study cohort

In an initial evaluation,20 DBS specimens collected from patients with confirmed PCD were used to assess the robustness of the iPLEX assay. From January 2017 to December 2018, 204,777 newborns were screened via MS/MS at Quanzhou Maternity and Children’s Hospital; newborns with low C0 levels (C0 < 8 μmol/L, cut-off value: 8–50 μmol/L) were recruited for the study. This study was approved by the Ethical Committee of Quanzhou Maternity and Children’s Hospital. Written informed consent was obtained from the parents of all infants for the collection of DBS samples and the publication of medical data.

### Multiplex primary carnitine deficiency assay design and genotyping

PCR and iPLEX extension primers were designed for 17 *SLC22A5* mutations using MassArray Assay Design 3.1 software (Agena, San Diego, CA),with amplicon lengths of 80–120 bp and a mass range of 4,500–9,000 Da. The designed primers were run through BLAT and modified when necessary to avoid pseudogene amplification. The designed primers cover nearly all the most common *SLC22A5* gene mutation sites, based on the mutation frequencies reported in previous studies [5, 9, 24, 26, 28, 29] and a local mutation database (Additional file 1: Table S1). Genotyping was performed using the MassARRAY platform according to the manufacturer’s instructions.

### Screening algorithm and diagnostic evaluation

**Fig. 1:**
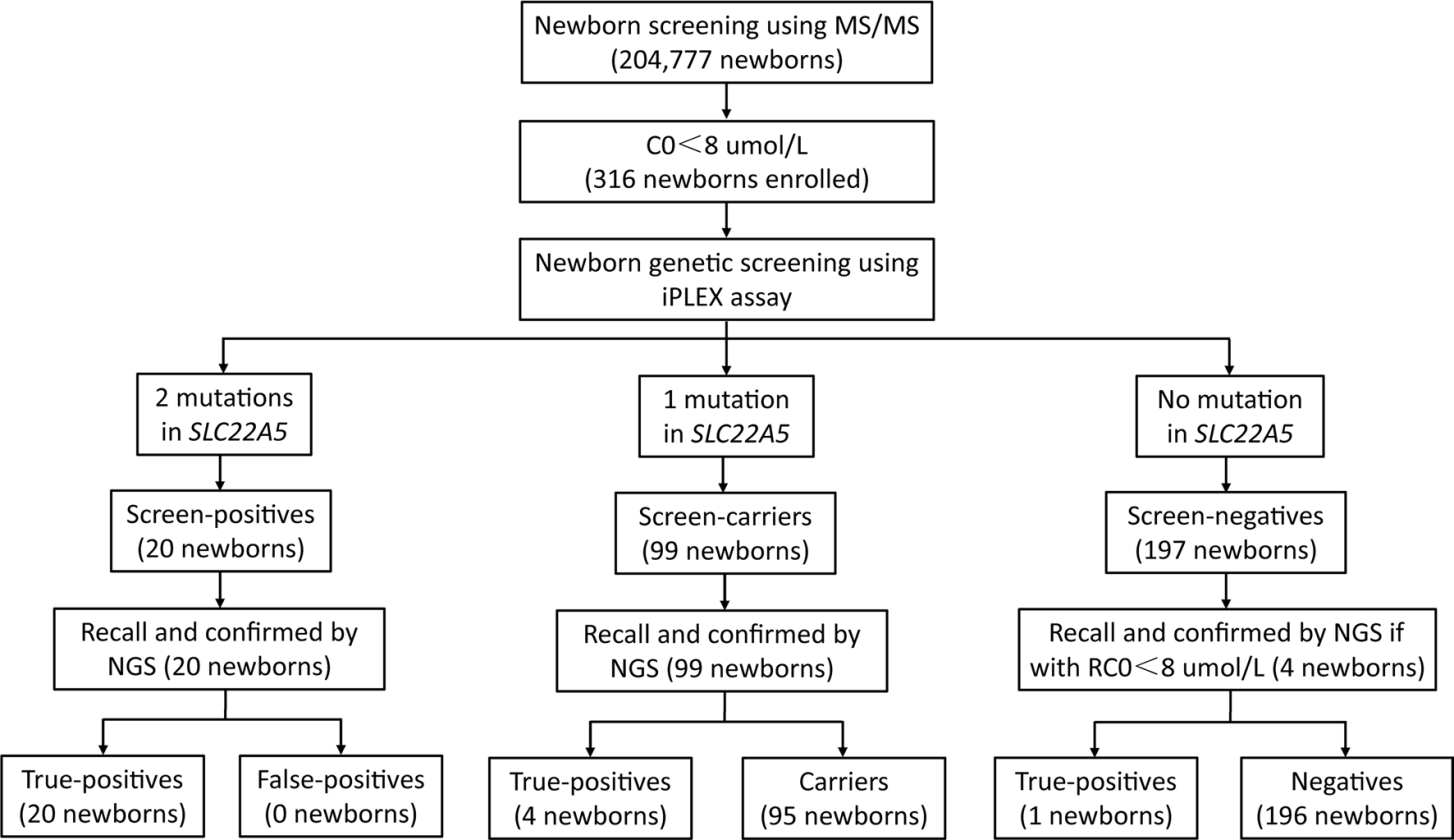
Theanalytical workflow of newborn second-tier genetic screening for primary carnitine deficiency (PCD). MS/MS indicates tandem mass spectrometry; NGS: next-generation sequencing; C0: free carnitine; RC0: retested C0 during the recall stage

A second-tier newborn genetic screening targeting17 *SLC22A5* mutations was performed, and the analytical workflow is summarised in Fig. 1. The C0 cut-off value was initially set to the 99.5th (0.05th) percentile in our laboratory but was slightly modified to minimise false-positive and false-negative results. After routine newborn metabolic screening was completed, DBS specimens collected from newborns with low C0 levels were sent for *SLC22A5* mutation screening. Newborns with two, one, and no *SLC22A5* mutations were defined as screen-positives, screen-carriers, and screen-negatives, respectively. All screen-positives and screen-carriers were recalled and underwent further diagnostic evaluation, including biochemical laboratory tests and genetic analysis. Screen-negatives were also recalled if their C0 levels were persistently low (i.e., below the cut-off in both the first and second screening), even if no mutations were identified, and were referred for diagnostic evaluation. Targeted next-generation sequencing (NGS) using an inherited metabolic disorder panel was performed as previously described [30]. A confirmatory diagnosis was made based on the presence of biallelic pathogenic variants in *SLC22A5*. Diagnosed patients were treated with L-carnitine supplementation (50–100 mg/kg/d).

## Supplementary Information

**Additional file1: Table S1.** Customized panels based on reported *SLC22A5* mutations.

## Data Availability

The datasets used and/or analysed during the current study can be obtained from the corresponding author upon a reasonable request.

## Abbreviations

PCD: Primary carnitine deficiency
OCTN2: Organic cation transporter type 2
MS/MS: Tandem mass spectrometry
NBS: Newborn screening
C0: Free carnitine
DBS: Dried blood spot
NGS: Next-generation sequencing
