# Supplementary material for: Increased detection of primary carnitine deficiency through second-tier newborn genetic screening
Source: Orphanet J Rare Dis. 2021 Mar 23;16:149. doi: 10.1186/s13023-021-01785-6 (PMC7988980; doi:10.1186/s13023-021-01785-6)
Supplement: Supplementary file 1 — Additional file1: Table S1. Customized panels based on reported SLC22A5 mutations. [file 13023_2021_1785_MOESM1_ESM.docx]

**Supplementary file 1: Table S1.** Customized panels based on reported *SLC22A5* mutations

| No. | Location | Nucleotide Change | Amino Acid Change | References |
| --- | --- | --- | --- | --- |
| 1 | Exon 4 | c.760C > T | p.R254X | Tang et al., (2002) |
| 2 | Exon 8 | c.1400C > G | p.S467C | Koizumi et al., (1999) |
| 3 | Exon 1 | c.51C > G | p.F17L | Lee et al., (2010) |
| 4 | Exon 4 | c.695C > T | p.T232M | Dobrowolski et al., (2005) |
| 5 | Exon 4 | c.797C > T | p.P266L | Chen et al., (2013) |
| 6 | Exon 7 | c.1139C > T | p.A380V | Chen et al., (2013) |
| 7 | Exon 1 | c.338G > A | p.C113Y | Han et al., (2014) |
| 8 | Exon 7 | c.1195C > T | p.R399W | EI-Hattab et al., (2010) |
| 9 | Exon 2 | c.428C > T | p.P143L | Lee et al., (2010) |
| 10 | Intron 2 | c.497+1G > T | - | Han et al., (2014) |
| 11 | Exon 3 | c.505C > T | p.R169W | Wang et al., (1999) |
| 12 | Exon 5 | c.865C > T | p.R289X | Dobrowolski et al., (2005) |
| 13 | Exon 8 | c.1445A > G | p.Y482C | Lin et al., (2020) |
| 14 | Exon 1 | c.95A > G | p.N32S | Rasmussen et al., (2014) |
| 15 | Exon 3 | c.517delC | p.L173Cfs | Han et al., (2014) |
| 16 | Intron 4 | c.824+1G > A | - | Zhang et al., (2019) |
| 17 | Exon 6 | c.976C > T | p.Q326X | This study |
